# Opportunistic Cervical Cancer Screening During Antenatal Visits: A Small Step Toward Early Detection

**DOI:** 10.7759/cureus.89433

**Published:** 2025-08-05

**Authors:** Ruchi Verma, Ritu Sharma, Pinky Mishra, Neha Mishra

**Affiliations:** 1 Obstetrics and Gynaecology, Government Institute of Medical Sciences, Greater Noida, IND; 2 Obstetrics and Gynaecology, Atal Bihari Vajpayee Institute of Medical Sciences and Dr. Ram Manohar Lohia Hospital, New Delhi, IND; 3 Obstetrics and Gynaecology, All India Institute of Medical Sciences, New Delhi, New Delhi, IND

**Keywords:** antenatal period, cervical cancer screening, co-testing, cytology, hpv dna

## Abstract

Background

Cervical cancer remains a significant public health concern in India. The objective of this study was to compare cytological abnormalities and HPV positivity rates between pregnant and non-pregnant women.

Materials and methods

This prospective observational study was performed at a tertiary care center in North India. A total of 120 women were included in the study, with 60 in each group. Group A included women who were pregnant, and Group B included non-pregnant women. Conventional Pap smear and co-testing (liquid-based cytology (LBC) and human papillomavirus deoxyribonucleic acid (HPV DNA)) were done on all the participants. The smears were interpreted according to the Bethesda system of classification. The results in the two groups were compared. Follow-up and management were done as per standard guidelines.

Results

Cervical smear was satisfactory in 112 (93.3%) and 116 (96.7%) of women who underwent conventional Pap smear and LBC, respectively. Among the premalignant lesions in Group A, atypical squamous cells of undetermined significance (ASC-US) were found in four (6.67%) of patients, and low-grade intraepithelial lesion (LSIL) was found in two (3.33%) of patients. In Group B, ASC-US was seen in five (8.33%), LSIL in three (5%), and high-grade squamous intraepithelial lesion (HSIL) in one (1.6%) case, respectively. The women in both groups who tested positive for HPV DNA were retested as per guidelines.

Conclusions

The prevalence of cytological abnormalities and HPV DNA positivity was corroborated well in both pregnant and non-pregnant women. Due to the increased number of antenatal visits and institutional deliveries, we would like to emphasize that screening for cervical cancer can be done by either HPV DNA or cytology in the antenatal period.

## Introduction

Cervical cancer accounts for the fourth position in terms of the most frequently diagnosed and common cause of death in women worldwide [1]. The main cause of cervical cancer is persistent infection with high-risk HPV, along with several co-factors like multiple sexual partners, sexually transmitted infections, positive family history, etc [2]. Cervical cancer has a long pre-invasive period of around 10-15 years, which gives a good opportunity for early detection and treatment in its initial pre-invasive phase.

In 2023, India witnessed 27,526 new cervical cancer cases and 79,906 deaths. The age-standardized mortality rate for cervical cancer was 11.2 per 100,000 women in 2023, which was significantly greater than the world average. As per prediction models, a total of 2,194,998 women in India will die from the disease between 2020 and 2070, which will further rise to 26,66,436 by 2120. The implementation of the WHO’s proposed "90-70-90" targets by 2030 would lead to the elimination of cervical cancer as a public health problem [3]. The potential of screening in India is immense, as there are 272.8 million eligible women [4]. 

As per 2018 data, the overall screening coverage is just 3.1% in India, much lower than the WHO’s global aim of 70% coverage, consisting of at least one screening between the ages of 30-45 years. Even in the regions having higher literacy rates, like South India, the screening coverage is just 7.1% with cytology as a primary screening tool [5-7]. To achieve the WHO’s screening target, the idea of utilizing antenatal periods seems practical. Most women in India go to an obstetrician for the first time when they are pregnant [8]. They are more receptive to medical advice and follow-up during this period. During pregnancy, the cervix enlarges, becomes soft and congested, is covered with copious mucus, and the columnar epithelium becomes everted and hypertrophied. The early second trimester is thus an ideal period as endocervical cells are translocated outside the cervix, the transformation zone is visualized easily, and sampling is easier currently without much cervical manipulation. It also allows screening for other infections as well. The endocervical sampler used in conventional Pap smear extends only to the lower half of the cervix, and various studies have shown its safety during pregnancy [9].

HPV vaccination coverage is also low in India, with less than 1% of girls [10]. However, the goal is to reach 90% by a certain period by introducing it in the national immunization program.

The objective of the present study was to compare cytological abnormalities and HPV positivity rates between pregnant and non-pregnant women.

## Materials and methods

This is a prospective observational cohort study that was conducted for a period of one year at Motilal Nehru Medical College, Prayagraj, India, from January 2014 to December 2015. We included pregnant and non-pregnant women attending the outpatient department. Women aged 25 to 40 years who were sexually active and willing to participate were included in the study, while those with a known diagnosis of cervical carcinoma, who were not sexually active, or who had active vaginal bleeding were excluded.

Ethical clearance was taken from the institutional ethical committee (IEC/MLNMC/2014/No. 5) before the recruitment of participants. The convenience sampling method was used. A total of 200 sexually active women of reproductive age who fulfilled the inclusion criteria were screened, enrolled, and given the option of opting for screening methods.

**Figure 1:**
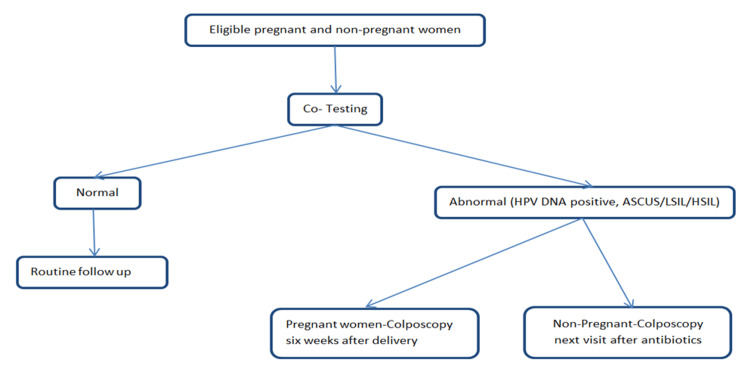
Flowchart of the study population HPV DNA: human papillomavirus deoxyribonucleic acid; ASCUS: atypical squamous cells of undetermined significance; LSIL: low-grade squamous intraepithelial lesion; HSIL: high-grade squamous intraepithelial lesion

Of these, 120 women opted for co-testing, with 60 in each group (Group A: pregnant; Group B: non-pregnant) (Figure 1). 

A detailed proforma was filled out after obtaining consent, including basic history, including family, personal, and surgical history, and chief complaints. A gynaecological examination was done for each patient. The pregnant women also underwent obstetrical examination. At the time of examination, samples for conventional pap smear, liquid-based cytology, and HPV testing were taken from all the patients.

An unlubricated speculum (saline or warm water may be used) was inserted to visualise the cervix and scrape material with the spatula from the whole circumference of the cervix. The Ayer’s spatula was withdrawn, and the cervicovaginal fluid was quickly collected, evenly spread onto a glass slide, and immediately fixed. After that, the endocervical brush was inserted into the endocervical canal and rotated half a turn. The endocervical brush was withdrawn, and the collected cervicovaginal secretion was spread quickly and evenly onto the second slide and fixed immediately (drop slide into fixative or spray with fixative, holding the spray bottle approximately 8-12 inches from the slide). The first and second slides were used for a Pap smear. The Papanicolaou staining procedure was used, employing hematoxylin as a nuclear stain and Orange G-6 and Eosin Alcohol (EA-36) as cytoplasmic counterstains. The slides are finally mounted in Canada balsam, examined under a microscope, and interpreted by a pathologist.

For the co-testing, the third sample was taken with the help of a broom-type cyto-brush in the same manner, and a self-detachable brush was placed in the vial of liquid preservative. With this technique, 80-90% of the cells are transferred to the liquid media, as compared to only 10-20% transferred via conventional Pap smear [11]. The cells are retrieved from the vial by passing the liquid through a filter, which traps the larger epithelial cells, separating them from the small blood and inflammatory cells. This process leads to a thin layer of diagnostic cells properly preserved and more easily interpreted by the cytologist. HPV DNA detection was done by polymerase chain reaction, which is an amplification method. The interpretation of findings was done as per the Bethesda System, where specimen adequacy is classified as a minimum of 8,000 to 12,000 cells(conventional preparation) and 5,000 cells (liquid-based cytology). If more than 75% of cells are obscured due to inflammation, bacteria, or lubricants/blood, the smear is categorised as unsatisfactory [11].

The outcome measures were cytological abnormalities and HPV positivity rates between pregnant and non-pregnant women. 

Statistical analysis

SPSS version 21 (IBM Corp., Armonk, NY) was used for the statistical evaluation of our research data. The categorical variables, such as socioeconomic profile, findings on cytology, and HPV status, were reported as numbers and percentages. Continuous variables, such as age and duration of marriage, were reported as means and standard deviations. The chi-square test was used to compare the differences between groups for categorical variables, while the t-test was used for continuous variables. A p-value of <0.05 was considered statistically significant.

## Results

A total of 120 women were recruited in the study, with 60 in each group. The distribution of demographic variables and cytological findings is shown in Table 1. The cervical smear was satisfactory in 112 (93.3%) and 116 (96.7%) out of 120 patients in the group undergoing conventional Pap smear and LBC, respectively. In Group A, atypical squamous cells of undetermined significance (ASCUS) were found in four (6.67%) patients, and low-grade intraepithelial lesion (LSIL) was found in two (3.33%) patients (Table 1).

**Table 1:** Demographic variables and cytological findings n (%): number (percentage); ASCUS: atypical squamous cells of undetermined significance; ASC-H: atypical squamous cells – high-grade squamous intraepithelial lesion; LSIL: low-grade squamous intraepithelial lesion; HSIL: high-grade squamous intraepithelial lesion; HPV: human papillomavirus. *chi-square test has been used for categorical variables

Parameter	Pregnant women; Group A (n=60)		Non-pregnant women; Group B (n=60)	P-value^*^
Age (mean±SD)	31.2±3.7		31.1±3.4	0.4
Socioeconomic profile; n(%)				
Upper class	14(23.3)		9(15.0)	0.3
Middle class	21(35.0)		22(36.6)	
Lower class	25(41.6)		29(48.3)	
Duration of marriage in years; n(%)				
<5	12(20.0)		26(43.3)	0.1
5-10	32(53.3)		19(31.6)	
10-15	10(16.6)		10(16.6)	
>15	6(10.0)		5(8.3)	
Findings on cytology; n(%)				
Normal		11(18.33)	10(16.66)	0.8
Infections		18(30.00)	23(38.33)	0.3
Inflammation		25(41.66)	18(30.00)	0.2
ASCUS/ASC-H		4(6.67)	5(8.33)	0.7
LSIL		2(3.33)	3(5.00)	0.7
HSIL		0	1(1.66)	0.3
HPV status		10(16.7%)	8(13.3%)	

The prevalence of HPV positivity in patients with abnormal cytology was 12.5%. Colposcopy was performed in all HPV-positive patients. The results were compared with cytology and colposcopy findings between both groups (Tables 2-3).

**Table 2:** Cytology and colposcopic biopsy findings of HPV-positive patients in Group A (pregnant women) ASCUS: atypical squamous cells of undetermined significance; LSIL: low-grade squamous intraepithelial lesion; HPV: human papillomavirus; CIN: cervical intraepithelial neoplasia

HPV+ve, n=10	Cytology findings	Colposcopy and biopsy findings
Patient 1	Normal	Normal
Patient 2	ASCUS	Leucoplakia
Patient 3	Inflammation	Low grade
Patient 4	Infection	Normal
Patient 5	LSIL	CIN I
Patient 6	ASCUS	Normal
Patient 7	ASCUS	Normal
Patient 8	Infection	Normal
Patient 9	LSIL	CIN I
Patient 10	ASCUS	Lost to follow up

**Table 3:** Cytology and colposcopic biopsy findings of HPV-positive patients in Group B (Non-pregnant women) ASCUS: atypical squamous cells of undetermined significance; HSIL: high-grade squamous intraepithelial lesion; LSIL: low-grade squamous intraepithelial lesion; HPV: human papillomavirus; CIN: cervical intraepithelial neoplasia

HPV+ve, n=8	Cytology findings	Colposcopy and biopsy findings
Patient 1	HSIL	CIN II
Patient 2	ASCUS	Normal
Patient 3	ASCUS	Lost to follow up
Patient 4	ASCUS	Leucoplakia
Patient 5	LSIL	CIN I
Patient 6	LSIL	CIN I
Patient 7	HSIL	CIN I
Patient 8	ASCUS	Normal

Follow-up was done with co-testing as per standard guidelines. Two patients were lost to follow-up. Two antenatal women were found to be HPV DNA-positive in the postpartum period on follow-up. Five non-pregnant women were positive for HPV DNA on follow-up.

## Discussion

Since the cervical cancer vaccination for adolescent girls is yet to be rolled out as a national program, screening for cervical cancer remains the mainstay for preventing cervical cancer in India. The WHO recommendations on cervical cancer screening are visual inspection with acetic acid (VIA) in low-resource settings, cervical cytology (PAP smear) every three to five years, or HPV testing every five years [12,10]. However, despite many years of implementing VIA and cytology, India's screening coverage has not increased, and therefore, the prevalence and mortality of carcinoma have shown a very slow downward trend [13-14].

Evidence suggests that HPV DNA-based screening gives 70-80% greater protection compared to cytology [15]. HPV DNA is a highly sensitive test, and it just needs half the frequency of visits when compared to the conventional cytological tests. Ideally, cytology should be done every three years in women from 21-65 years, amounting to approximately 15 hospital visits, whereas on the other hand, HPV DNA-based screening allows the extension of the screening interval to five years. This is a feasible option in low-resource settings where screening by this highly sensitive test once or twice in the whole lifetime of a woman is sufficient [10,13,16]. The American Cancer Society endorses HPV DNA as the primary method of screening in reproductive-age women starting from the age of 25 years [17].

The implementation of organised screening in India continues to be difficult. Organising even one or two rounds of screening is associated with logistical difficulties in the health systems already overwhelmed by the demands of both infectious and non-communicable diseases [18].

Antenatal and postpartum care should include an evaluation of cervical cancer screening in pregnant and postpartum women. Screening should be offered to women with no history of screening. Screening is safe at any stage of pregnancy. American and Australian guidelines recommend a timely colposcopy for pregnant women who test positive for HPV. In cases that are screened with cytology triage, early colposcopy is recommended in cases of HSIL or glandular changes. Cases with CIN 2/3 can be followed postpartum or monitored with colposcopy every 12-24 weeks [12,19]. Canadian guidelines advocate repeating HPV-based screening three months postpartum for those with high-risk HPV and low-grade cytology (ASCUS/LSIL). Those with high-grade or glandular cytology (ASC-H, HSIL, AGC) should be referred for colposcopy earlier, i.e., within four weeks [20].

In our study, most women belong to a low socioeconomic status, having many risk factors for cervical cancer, such as age, multiparity, etc. Most of the women presented to the outpatient department with chief complaints of discharge per vaginum, similar to the findings reported by Koteswari et al. [21]. There was no significant difference in cytological findings reported between the two groups. This suggests that pregnant patients have findings similar to non-pregnant women. Hence, antenatal visits can be used as an opportunity for cervical cancer screening. Other authors have reported a comparison of cytopathological findings in pregnant women and non-pregnant women. ASCUS had a higher frequency in non-pregnant women. Similar findings were reported by Dufloth et al., who noted a higher frequency of ASCUS in non-pregnant women as compared to pregnant women [22]. Xavier-Júnior et al. demonstrated that the cytological prevalence of HSIL was similar in both pregnant and non-pregnant women, irrespective of age [5].

The prevalence of high-risk HPV is 8.2% in pregnancy [23]. In the present study, HPV positivity was comparable in the pregnant and non-pregnant study groups. HPV screening between 16-20 weeks of pregnancy is viable, practical, and can surely add to the cervical cancer screening rate in the country. Pregnancy is a good opportunity to improve awareness of the screening programmes. So, it is recommended that cervical cancer screening should be included in the routine antenatal care, as for many women, pregnancy is the first, or only, opportunity in the developing country for cervical screening in the current scenario [23].

The strengths of this study are that both the cytology and HPV DNA samples were obtained from both groups to ensure corroboration of findings, and the follow-up was ensured with very few losses. However, the limitation of the study was its modest sample size and the requirement for long-term follow-up in pregnant women.

## Conclusions

The prevalence of cytological abnormalities and HPV DNA positivity correlates well in both pregnant and non-pregnant women. Due to the increased number of antenatal visits and institutional deliveries, we would like to emphasize that either HPV screening or cytology may be used as a primary screening method in the antenatal period. Cervical cytology can be used in pregnant women where testing for HPV DNA is not available. The antenatal period should be used as an opportunity to raise awareness and improve the screening among young females. This can play a pivotal role in achieving the WHO target of 70% screening coverage among women and in advancing cervical cancer elimination within available resources.
